# Multidisciplinary Approach for the Management of Dubowitz Syndrome for Optimal Functional and Behavioral Outcomes: A Case Report and Brief Review of the Literature

**DOI:** 10.7759/cureus.76804

**Published:** 2025-01-02

**Authors:** Kenzo Alejandro Fukumoto Inukai, Braulio Ríos Muñoz, Carlos A Morales Morales, Lidia Mendoza Andrade, Cindy A Hernández Cárdenas, Leticia Sánchez Méndez, Rogelio Martínez Wagner

**Affiliations:** 1 Department of Plastic and Reconstructive Surgery, Palate/Lip Cleft Clinic, Hospital General Dr. Manuel Gea González, Mexico City, MEX

**Keywords:** autosomal recessive, cleft palate, craniofacial abnormalities, dubowitz syndrome, multidisciplinary treatment

## Abstract

Dubowitz syndrome (DubS) is a rare condition characterized by a range of medical challenges, including distinctive facial features and complications affecting the ocular, dental, dermatological, skeletal, cardiovascular, gastrointestinal, neurological, immunological, and hematological systems. This syndrome results from multiple gene mutations and is inherited in an autosomal recessive manner. The purpose of this article is to detail the multidisciplinary approach required to address the various clinical and social aspects of the syndrome. We present the case of a male Mexican patient diagnosed with DubS based on clinical features, particularly the characteristic facial appearance, despite inconclusive genetic testing. He displayed an average intellectual level but experienced behavioral issues, alongside ocular, dental, cutaneous, musculoskeletal, gastrointestinal, and hematological alterations. The patient received comprehensive care from a multidisciplinary team, including specialists in plastic and reconstructive surgery, pediatrics, psychology, genetics, nutrition, orthodontics, ophthalmology, stomatology, and phoniatrics. This collaborative approach resulted in positive functional and behavioral outcomes. Additionally, we conducted a review of the literature, noting that there are currently no established treatment guidelines. A thorough multidisciplinary strategy for patients with DubS can lead to improved esthetic and functional results, as well as enhanced social skills and self-esteem.

## Introduction

Dubowitz syndrome (DubS) is a rare autosomal recessive disorder first identified in 1965 by Victor Dubowitz [1]. The condition was later named and characterized by Grosse and Opitz [2]. The prevalence of DubS is not well-established in the literature. However, it has been estimated to occur in approximately one in 125,000 to one in 1,000,000 live births [3]. Over the past 60 years, approximately 200 cases have been documented worldwide. The diagnosis of this syndrome is challenging due to its wide spectrum of clinical manifestations and the lack of an established genetic basis. To date, no specific etiology has been identified, but it has been associated with chromosomal breakage abnormalities and different genes implicated [4-7]. 

This syndrome is characterized by a distinctive facial gesture, cognitive and behavioral issues, and growth and developmental abnormalities. It goes along with a range of abnormalities affecting the skin, eyes, teeth, urogenital system, musculoskeletal system, cardiovascular system, gastrointestinal tract, neurological functions, hematological parameters, and risk of malignancies [5,6,8]. Due to the diverse clinical manifestations of DubS, it can be confused with differential diagnoses such as Bloom syndrome (BSyn) and fetal alcohol syndrome. However, the distinctive facial features of DubS, along with a medical history and additional studies, help guide the clinical diagnosis [5,8]. Although DubS is well-documented in the medical literature, it is mainly represented by case reports and case series that address the management of specific clinical features, with limited discussion on a comprehensive multidisciplinary approach. The aim of this article is to offer recommendations for management and highlight the advantages of a multidisciplinary treatment strategy for enhancing functional and psychological development in affected patients.

Etiology 

The origins of DubS have not been fully elucidated. Reports of several families with affected siblings and unaffected parents, often within contexts of parental consanguinity, suggest that DubS is likely an autosomal recessive disorder [5-8]. There is documentation of one case involving vertical transmission [9] and another with a family history of DubS [10]. Recent advancements in genetic diagnostics have identified multiple genes associated with the clinical manifestations of this syndrome. The evidence suggests that DubS is not caused by a single gene mutation, indicating genetic heterogeneity. This may help explain the phenotypic variability observed in the syndrome [5-7]. Steward et al. [6] propose that DubS should not be considered a single condition but rather a group of disorders with similar phenotypic features. Dyment et al. [7] conducted exome or genome sequencing on 31 individuals clinically diagnosed with DubS and found that 45% of them had an alternative diagnosis. They proposed that the genes identified in their study might share common biological pathways, not only with each other but also with genes involved in single-gene disorders previously associated with DubS. This suggests that the DubS clinical phenotype may be linked to significant locus heterogeneity, with the molecular diagnoses corresponding to emerging clinical conditions that share overlapping features with the DubS phenotype.

Clinical features

The primary clinical manifestations of DubS include distinctive facial features accompanied by craniofacial anomalies [5,10,11]. Other common findings involve cutaneous [12], urogenital [8], musculoskeletal [10], ocular [13], and dental abnormalities [14,15]. Cognitive functioning and behavior are essential to be assessed [11]. Cognitive deficits range from normal intelligence to severe intellectual disability, and frequent behavioral issues such as aggressiveness, hyperactivity, shyness, and feeding problems [8,11,16]. Additionally, less common medical concerns have been linked to gastrointestinal, cardiovascular, hematological, and potential autoimmune disorders [5]. 

Children with DubS are at an elevated risk of developing a range of systemic neoplasms and represent one of the two causes of morbidity and mortality, along with bone marrow failure [5,8]. Malignancies are four times more common in women with DubS than in men with the condition [17]. Although no established statistics are available, malignancies have been reported in association with the syndrome, particularly acute lymphoblastic leukemia [18], lymphoma [19], esophageal squamous cell carcinoma [20], rhabdomyosarcoma [21], and neuroblastoma [19]. As part of the manifestations of bone marrow failure, fatal cases of aplastic anemia have been reported in patients with DubS [22]. As a result, the prognosis can be severe, significantly impacting the patient's lifespan [5,8].

Diagnosis

The clinical variability and low incidence of DubS present challenges for accurate diagnosis. A detailed medical and neurodevelopmental history, along with a physical examination that focuses on growth parameters and clinical features, is essential [5,11]. To suspect this condition, a classical phenotype must be identified, which includes mild intellectual disability, short stature, microcephaly, a sloping forehead, ptosis, telecanthus, eczema, and a high-pitched voice [11]. Consequently, molecular diagnosis is required to confirm the suspicion [4-7].

Exome and genome sequencing have shown that no single gene, cluster of associated genes, or shared pathway is responsible for the syndrome's etiology; rather, multiple genes are involved. The most commonly identified biallelic variants in large-scale genomic studies include SKIV2L, SLC35C1, BRCA1, and NSUN2. Additionally, de novo variants in ARID1B, ARID1A, CREBBP, POGZ, TAF1, and HDAC8 have been noted, along with copy-number variations at 1p36.11 (ARID1A), 8q22.2 (VPS13B), Xp22, and Xq13 (HDAC8) [7].

Clinical management

The focus of treatment should be on addressing the symptoms experienced by the patient to improve their quality of life. It should not be managed by a single specialist but rather through a multidisciplinary team that treats the affected systems or organs [5,23]. Common treatments may include multiple surgeries, medical consultations, medications, blood transfusions, growth hormone therapy, and appropriate nutritional plans tailored to the needs of patients with DubS [5,10,11,23,24]. It is crucial to identify which patients are at increased risk of malignancies or related complications through thorough medical history taking, which will help facilitate necessary screening examinations [17].

## Case presentation

**Figure 1:**
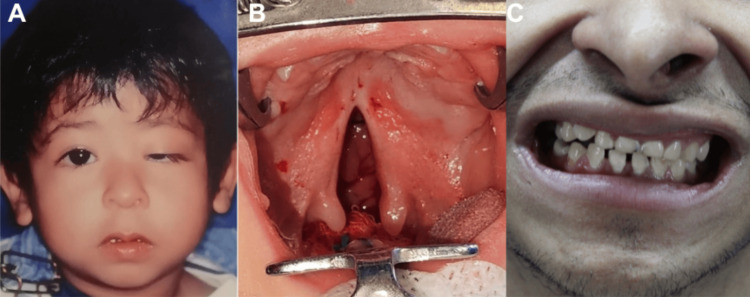
Preoperative clinical photograph of the patient (A) Clinical photograph of the patient with a facial appearance characterized by a triangular-shaped head, sloping forehead, flat supraorbital ridge, strabismus, congenital left palpebral ptosis, epicanthal folds, a broad and flat nasal bridge, a prominent round tip, long and flat philtrum, and micrognathia; (B) Preoperative photo of the cleft palate; (C) Preoperative frontal photo of dental anomalies, including oligodontia, fragile teeth, and dental hypoplasia.

A 23-year-old Mexican man presented to the craniofacial clinic in our center for follow-up. He was born to non-consanguineous Mexican parents, aged 24 and 30, after an uncomplicated full-term pregnancy. The patient had a low birth weight of 1,900 grams (-2.5 SD) and a length of 45 cm (-2 SD). At two years of age, he was evaluated by the pediatric team at our center, revealing several craniofacial features: microcephaly, a triangular-shaped head, a sloping forehead, a flat supraorbital ridge, strabismus, congenital left palpebral ptosis, epicanthal folds, a broad and flat nasal bridge, a prominent round tip, a long and flat philtrum, micrognathia, and cleft palate, as shown in Figures 1A-1B. At the same age, the patient was referred to the stomatology and orthodontics departments, where an intraoral examination revealed fragile teeth with poor enamel mineralization defects, as well as severe dental hypoplasia (Figure 1C). At that time, his weight was reported as 8,600 grams (-3 percentile), and his height was 76 cm (-3 percentile). He was referred to the nutrition service, where he was diagnosed with malnutrition. At age three, the patient was referred to the ophthalmologist for management of his strabismus. At age four, he was referred to our service for management of the cleft palate and left palpebral ptosis. Developmental assessment using the Denver Developmental Milestones [25] revealed no abnormalities in gross motor, fine motor, cognitive, or socioemotional development. However, a language delay was noted, likely due to the cleft palate. The patient was referred to speech therapy and phoniatrics following cleft palate repair. The patient experienced frequent gastroesophageal reflux from birth until the age of 12, which was managed by the nutrition department. Hematological studies showed no abnormalities in white blood cells or platelets, although he was diagnosed with asymptomatic grade I anemia based on the World Health Organization classification [26].

Regarding cognitive function, the patient was evaluated by a pediatric neuropsychologist at the age of four using the Terman-Merrill scale [27], which assesses intelligence across six areas: general intelligence, knowledge, fluid reasoning, quantitative reasoning, visual-spatial processing, and working memory. The overall result was within the average intellectual range. Behavioral studies through observation revealed that the patient was sociable, talkative, and friendly. Contrary to common reports in the literature, he did not display hyperactivity, aggression, or shyness. However, he did report issues with concentration. He had a deep passion for music and dancing, which were his primary hobbies. The patient also faced significant emotional challenges, including low self-esteem, largely due to bullying at school. His peers mocked him for his short stature, distinctive facial features, and high-pitched voice. Additionally, his relationship with his father was strained, as his father had rejected him since birth due to his condition. His primary support network consisted of his mother and sister. As a result, the patient underwent psychological therapy at age nine, which included a series of projective tests such as the Machover test (the human figure test) [28] and the Family Drawing test [29]. These assessments revealed the patient's struggles with self-esteem and his troubled relationship with his father. Importantly, no urogenital, cardiovascular, or immunological issues were identified.

The primary diagnosis was DubS, and the patient was referred to the genetics department for further evaluation. Differential diagnoses included the Pierre Robin sequence due to the presence of micrognathia and cleft palate. However, the absence of glossoptosis and airway obstruction ruled out this condition, and genetic testing was inconclusive for the Pierre Robin sequence. Bloom syndrome was also considered, based on clinical features such as microcephaly, a narrow head shape, micrognathia, and growth delays. However, the absence of malar telangiectasia, photosensitivity, café-au-lait macules, and immunodeficiency, along with a normal karyotype (46, XY), led to the exclusion of this diagnosis. Fetal alcohol syndrome was another consideration, based on features such as microcephaly, epicanthal folds, and a flat nasal bridge. However, the patient’s mother denied any alcohol consumption during pregnancy, which excluded this diagnosis.

Although genetic testing for DubS was inconclusive, the diagnosis was ultimately confirmed based on the characteristic facial features. Table 1 outlines the most common genetic mutations associated with this condition, and Table 2 provides a summary of the clinical features of DubS as reported in the literature.

**Table 1:** Affected gene in Dubowitz syndrome and the mechanism of mutation HDAC: histone deacetylase; NSUN2: nucleolar SUn-domain containing 2; VPS13B: vacuolar protein sorting 13 homolog B; POGZ: pogo transposable element derived with ZNF domain; TAF1: TATA-box binding protein-associated factor 1; BRCA1: breast cancer 1; CREBBP: CREB binding protein; SLC35C1: solute carrier family 35 member C1; ARID1B: AT-rich interaction domain 1B; SKIV2L: SKI family RNA helicase 2-like Sources: [4–7]

Gene	Etiology	Inheritance
HDAC8	De novo X-linked mutations	X-linked de novo
NSUN2	Pathogenic biallelic mutations	Autosomal recessive (compound heterozygous)
VPS13B	Pathogenic biallelic mutations	Autosomal recessive
POGZ	De novo and inherited dominant mutations	De novo dominant
TAF1	De novo X-linked mutations	X-linked de novo
BRCA1	Pathogenic biallelic mutations	Autosomal recessive (compound heterozygous)
CREBBP	De novo and inherited dominant mutations	De novo dominant
SLC35C1	Pathogenic biallelic mutations	Autosomal recessive (homozygous)
ARID1B	De novo and inherited dominant mutations	De novo dominant
SKIV2L	Pathogenic biallelic mutations	Autosomal recessive (homozygous)

**Table 2:** Range of clinical features of Dubowitz syndrome Sources:  [3–5, 9–23]

Manifestations of Dubowitz syndrome	
Craniofacial	Microcephaly, craniosynostosis, narrow or triangular-shaped head, sloping forehead, narrow bifrontal diameter, cleft palate, submucous cleft palate, and cleft uvula; flat supraorbital ridge; abnormally low-set prominent ears; long nose; broad and flat nasal bridge; prominent round tip; long and flat philtrum; micrognathia and prognathism.
Dental	Crowded and irregular teeth, delayed eruption, microdontia, taurodontia, anodontia/hypodontia or hyperdontia
Eyes	Hyperopia, nystagmus, cataracts, tapetoretinal degeneration, strabismus, blepharophimosis, palpebral ptosis, excessive epicanthal folds, telecanthus, hypertelorism, hypotelorism, microphthalmia, iris coloboma, and scanty lateral eyebrows
Cognition	Range from normal intelligence to severe retardation. Problems with spatial perception, numerical abilities, spelling, abstract reasoning, visual motor coordination, and fine motor development.
Behavioral	Hyperactivity, short attention span, impulsivity, aggressiveness, shyness, sensitivity to criticism, and refusal of food. A preference for vibrations and musical beats
Growth	Microcephaly, short stature, and low weight
Musculoskeletal	Partial webbing of the fingers and toes, sacral dimple, scoliosis, fifth finger clinodactyly, second-third toe syndactyly, footedness, clubfoot, dorsiflexion, plantar flexion, abducted and inverted foot, pectus excavatum, joint hyperextensibility, and pes planovalgus
Skin	Eczema, dermatitis, sparse and thin hair, and pyoderma gangrenosum
Urogenital	Hypospadias, cryptorchidism, small testes, and hypoplastic genitalia
Gastrointestinal	Chronic diarrhea, constipation, rectal prolapse, anal stenosis, congenital constipation, inguinal and hiatal hernia, and achalasia
Cardiovascular	Ventricular septal defects, patent ductus arteriosus, atrial septal defect, mitral valve prolapse
Hematologic	Blood dyscrasias (white, red, pancytopenia), aplastic anemia, IgA deficiency, hyper-IgE, hypogammaglobulinemia
Malignancy	Breast cancer, acute lymphocytic leukemia, non-Hodgkin’s lymphoma, esophageal squamous cell carcinoma, rhabdomyosarcoma, neuroblastomas

A multidisciplinary team at Gea González Hospital in Mexico City, Mexico, consisting of specialists in pediatrics, genetics, plastic surgery, ophthalmology, stomatology, orthodontics, nutrition, speech therapy, and phoniatrics, was assembled to address the complex and diverse needs of the patient. Recommended evaluations following diagnosis are summarized in Table 3.

**Table 3:** Suggested assessments after diagnosis of Dubowitz syndrome Sources: [3,5,11,13,14,16,17,23]

System/Concern	Evaluation	Comment
Feeding/Nutrition/Gastrointestinal	Consult with a gastroenterologist and feeding specialist	Evaluate for gastroesophageal reflux, malnutrition, and growth retardation; regular monitoring of weight, height, and nutritional intake is essential.
Pediatrician	Comprehensive pediatric evaluation	Regular health check-ups to monitor growth and development; screening for developmental delays and potential comorbidities (e.g., blood dyscrasias, aplastic anemia, and malignancies).
Ophthalmology	Consultation with ophthalmologist	Screen for strabismus, congenital ptosis, cataracts, and other ocular anomalies
Dermatology	Detailed skin exam	Evaluate for eczema, fine hair, photosensitivity rash, telangiectasia, and more severe conditions such as pyoderma gangrenosum
Immune system	Immunological screening (immunoglobulin levels, B/T lymphocytes, antibody responses)	Regular monitoring for recurrent infections
Phoniatrics/Speech therapy	Speech and language assessment	Comprehensive therapy for speech delay, language development, and articulation issues. Address cognitive and phonological challenges.
Cancer screening	Screening tests (e.g., abdominal ultrasound, whole-body MRI, breast MRI, blood tests, molecular testing)	Early detection of cancers (breast cancer, acute lymphocytic leukemia, non-Hodgkin’s lymphoma, esophageal squamous cell carcinoma, rhabdomyosarcoma, neuroblastomas)
Genetic counseling	Referral to genetics professionals for genetic counseling.	Inform the family about the nature and inheritance pattern of DubS, aiding in medical and personal decision-making.
Plastic surgeon	Surgical consultation with plastic surgeons	Surgical management of cleft palate, ptosis, musculoskeletal alterations, and aesthetic procedures
Dental care	Routine dental check-ups and preventive care	Comprehensive dental care (regular dental monitoring is essential); the primary teeth tend to remain for more prolonged periods.
Neurological assessment	Cognitive and developmental assessments	Cognitive deficits range from normal intelligence to severe intellectual disability. It can guide treatment and individualized education plans
Neurobehavioral/Psychosocial Care	Referral to psychological services for emotional and behavioral support	Early intervention for aggressiveness, difficulty concentrating, sleep disturbance, and feeding problems, hyperactivity, low self-esteem, bullying, and strained family relationships.

**Figure 2:**
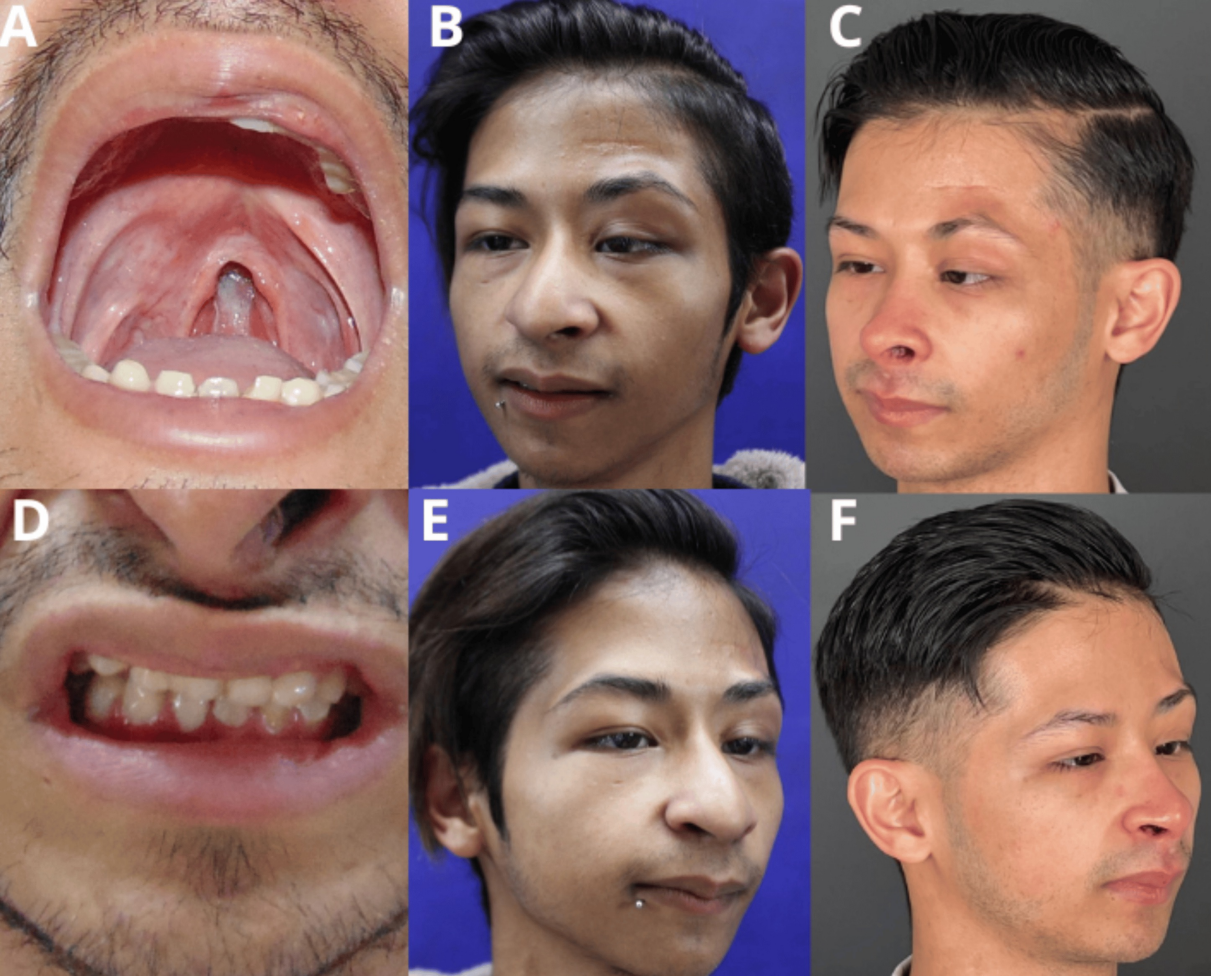
Postoperative clinical photographs of the patient A: Frontal photo of the patient after palatoplasty; the cleft palate is closed without fistulas; B and C: Frontal photo of the patient after autologous forehead lipoinjection, the forehead and left superciliary region have an aesthetically pleasing appearance, with left palpebral ptosis and strabismus corrected; D: The patient's teeth after orthodontic and stomatological treatment; E and F: Following rhinoplasty, the nose has a narrow base and tip, showing no deviation and symmetrical nostrils.

At age four, a palatoplasty was performed by our team using a minimal incision technique for a Group I cleft palate (Mendoza’s Classification) (Figure 2A). At the same age, the patient was referred to the speech therapy department. Upon evaluation, no hypernasality or nasal voice emission was detected; however, the patient exhibited a telegraphic speech pattern with simple sentences, poor linguistic organization, delayed language acquisition, and multiple unresolved phonological processes. These included dyslalia in monosyllabic and heterosyllabic groups for the phonemes |R|, distortion of the |S| phoneme, and compensatory articulations due to the recently repaired cleft palate. The patient received speech therapy for two and a half years with weekly sessions. The therapy was comprehensive, addressing various areas of language and communication, including metacognitive strategies to improve linguistic organization, articulation exercises, phonetic awareness modeling, and word and sentence completion exercises for unresolved phonological processes. At age five, the plastic surgery department performed frontalis suspension surgery to repair congenital left palpebral ptosis, and the ophthalmologist performed strabismus surgery (Figures 2B-2C). The stomatology and orthodontic departments treated enamel mineralization defects using a 5% fluoride varnish (sodium fluoride, 22,600 ppm) as a preventive measure against dental caries. For dental hypoplasia, type II glass ionomer cement and resin-based restorations were applied. Pulpotomies were performed to manage caries, and amalgam dome restorations were placed on fragile teeth. Multiple dental extractions were required throughout his development due to the delay in the eruption of the permanent teeth (Figure 2D). At age 18, elective rhinoplasty was performed to address anatomical concerns, including a wide nasal base, a prominent round tip, and asymmetrical nostrils (Figures 2E-2F). Two months later, the orthodontic department recommended the use of a nose shaper to manage left nostril collapse post-rhinoplasty. At age 22, to improve the aesthetic appearance of the left side of the forehead, liposuction and autologous lipoinjection were performed (Figure 2E-2F). The management of the nutrition department initially involved calculating the total energy expenditure and adding 10-20% to account for growth-related issues. The treatment plan included a high-calorie diet, predominantly composed of carbohydrates and lipids, along with increased-calorie-density formulas and recommendations to avoid irritant foods and measures to alleviate gastroesophageal reflux symptoms. The patient continues to be monitored by the nutrition department every three months. Currently, the patient weighs 40 kilograms, has a height of 1.47 meters, and has a body mass index (BMI) of 18.5 kg/m^2^. The current dietary plan consists of 2,500 kcal per day, with 55% from carbohydrates, 30% from lipids, and 15% from proteins.

Due to bullying at school and a strained relationship with his father, the patient began psychological therapy at the age of nine, receiving weekly sessions based on a cognitive-behavioral approach. This therapy, which continued until the age of 14, played a crucial role in emotional regulation and in addressing the patient’s low self-esteem and concentration difficulties.

The patient is currently 23 years old and continues to receive follow-up care from the nutrition, dentistry, stomatology, and plastic surgery departments. Nutritionally, the patient has a BMI within the normal range. In terms of language, the patient demonstrates proficiency in complex sentence construction, good linguistic organization, and no compensatory articulations or dyslalia, with no signs of velopharyngeal insufficiency or oropharyngeal fistulas. The patient also maintains good dental health and has no feeding issues. The left ocular opening is normal. A postoperative satisfaction scale, used at our center, was applied to assess the results of rhinoplasty and forehead surgery, with the patient rating both procedures a score of four (satisfied, but needs minor adjustments; Appendix 1). The patient expressed happiness with the aesthetic outcomes. Currently, the patient is pursuing a degree in programming and holds a job, performing both tasks without concentration-related issues. The patient reports good self-esteem and has learned to adapt to and cope with challenging social situations in daily life, including those in academic, work, and family settings.

The patient provided a signed informed consent statement approving the use of his photographs in this case report.

## Discussion

The Gea Hospital in Mexico City is a leading reference center for managing rare pediatric syndromes, including those with craniofacial anomalies, through a multidisciplinary approach. Dubowitz Syndrome is extremely rare, with fewer than 200 cases reported globally and only one in Latin America [23]. There are no established guidelines for managing its diverse clinical features, and early diagnosis is challenging due to its low prevalence and variable manifestations.

The first major study of DubS was a review of 141 patients published by Tsukahara and Opitz in 1996 [8], which described a broad spectrum of clinical features associated with DubS. According to multiple studies, this syndrome should be suspected in newborns who exhibit characteristics such as intrauterine growth restriction and distinct facial features, including a triangular facial shape, sparse hair and eyebrows, a sloped forehead, small palpebral fissures, bilateral ptosis, epicanthic folds, a flat nasal bridge, a broad nasal root, anteverted nares, abnormally shaped ears, and a high palate with potential submucous cleft palate and micrognathia. Additionally, affected individuals may experience growth retardation, mild to moderate intellectual disability, microcephaly, eczema, behavioral issues, and a distinctive high-pitched voice [1,5,6,8,12,14].

In our case, although genetic testing did not conclusively confirm the diagnosis, the characteristic facial features were instrumental in clinical evaluation for the diagnosis. A study by Innes et al. [5] indicated that genetic causes were identified in only 33% of clinically diagnosed DubS cases, suggesting that facial anomalies remain the most reliable diagnostic indicators despite genetic heterogeneity.

A comprehensive multidisciplinary treatment strategy is essential for managing DubS, necessitating collaboration among specialists such as plastic surgeons, pediatricians, psychologists, geneticists, nutritionists, dermatologists, orthodontists, ophthalmologists, and speech therapists. This approach helps deliver individualized care tailored to the unique challenges faced by each patient [24]. As noted by Korylchuk et al. [30], a multidisciplinary approach in clinical medicine has been shown to contribute to reduced mortality, complications, length of hospitalization, and readmissions while also enhancing patient satisfaction. The fragmented healthcare system with no communication between professionals is one of the major barriers to its implementation [30]. Another significant obstacle is the lack of time in each medical consultation to provide comprehensive care when the patient is seen by multiple services [30]. The Cleft Lip and Palate Service at our center, led by the senior author Dr. Martínez Wagner Rogelio, addresses these challenges by integrating the multidisciplinary team (pediatrician, plastic surgeon, rehabilitation specialist, orthodontist, speech therapist, and psychologist) into each consultation. This model allows specialists from various fields to collaborate in the same room, ensuring holistic, coordinated care. After each consultation, the team reviews and adjusts the treatment plan to provide comprehensive, high-quality care.

One of the main differential diagnoses for DubS is BSyn, an autosomal recessive disorder that shares many clinical features with DubS, and therefore, its management is similar. Langer et al. [24], in their study on BSyn, emphasize the need for a multidisciplinary approach to address each manifestation of this syndrome. As DubS, the clinical diagnosis of BSyn should be confirmed by genetic professionals [24]. Growth retardation and feeding issues are the most consistent clinical features of BSyn, where a nutritionist should prescribe formula and nutritional supplements with increased caloric density during infancy and childhood, as in our patient, along with close monitoring of growth and weight gain. Gastroesophageal reflux is also common in BSyn, requiring evaluation by a gastroenterologist and implementation of anti-reflux measures. Skin involvement, such as the appearance of a sun-sensitive rash on the nose and cheeks, poikiloderma, and telangiectasias, needs to be managed by a dermatologist [24]. Although our patient did not exhibit dermatological lesions, skin manifestations have been reported in DubS. Tsukahara and Opitz [8] documented fine hair and eczema as the most frequent cutaneous features in their series of cases, with 58 and 59 cases, respectively. Dieter Vieluf et al. [12]. reported a two-year-old male with DubS who developed erythema, reddish papules, and lichenification on the front of his legs, which cleared completely with the use of topical steroids (prednicarbate). More severe skin manifestations, such as pyoderma gangrenosum and its management, have also been described in DubS by Ghode et al. [3], who emphasized the need for care by a dermatologist and a multidisciplinary team.

Laboratory evaluation of the immune system should be addressed in both syndromes [24]. In BSyn, IgM and IgA levels are most commonly affected, resulting in a higher risk of opportunistic infections [24]. Although less common, immune deficiencies such as IgA deficiency, hyper-IgE-like syndrome, hypogammaglobulinemia, and profound T-cell defects have been reported in patients with DubS [4,9,19,20,31]. Sarmento Jr. et al. [9] reported the first case of DubS with hyper-IgE-like syndrome and nasal polyps, which responded well to low-dose steroids. Thuret et al. [4] described two patients with DubS who had repeated infections and recurrent ulcerative stomatitis, along with IgA deficiency, leukopenia, neutropenia, and agranulocytosis. Lougaris et al. [31] reported a patient with DubS and IgA deficiency, presenting significant T-cell defects similar to combined immunodeficiency, alongside a severe history of infections. Some cases of hematological and solid malignancies have been reported in patients with DubS associated with immune deficiencies or isolated. This highlights the need to assess the immune system and screen for malignancies in these patients. Turkbeyler et al. [20] reported a 24-year-old male with DubS who developed esophageal squamous cell carcinoma and IgA deficiency, with recurrent infections. He died four months after starting chemotherapy. Sauer and Spelger [19] reported two sisters with DubS, one diagnosed with neuroblastoma and hypogammaglobulinemia and the other with malignant lymphoma and IgA deficiency, who died after receiving radiotherapy. Additionally, Grobe [18] reported a patient with DubS who died of acute lymphocytic leukemia at age 6¾. Al-Nemri et al. [21] reported a patient with DubS born with a 10 × 15 cm mass over the right hemithorax, diagnosed as embryonal rhabdomyosarcoma, who died at three months of age. In this patient, genetic testing revealed multiple chromosomal breakages.

In DubS, dental and craniofacial abnormalities are primarily addressed through plastic and reconstructive surgery in conjunction with orthodontics and stomatology [14,23]. Plastic surgeons should play an active role in surgical procedures to treat craniofacial and musculoskeletal clinical features. For cleft palate, palatoplasty is recommended at an early age, as indicated by international guidelines, around nine to 12 months of age [32]; however, in our patient, this was performed at four years due to parental choice and growth delay. Likewise, elective rhinoplasty should be considered to improve both the functional and aesthetic appearance of the nose. It is up to surgical preference and philosophy whether early rhinoplasty should be made during palatoplasty or wait until osseous maturation. Our center does both; we approach alar and dorsal abnormalities during palate and oral surgery; however, we do not use any cartilage grafts or osteotomies during early intervention; we prefer to do a secondary procedure during adolescence (average 16 years old) to formal rhinoplasty. Ocular anomalies are common in these patients, necessitating intervention by the ophthalmology service. In our patient, the ophthalmology team performed strabismus correction and eyelid surgery using frontalis suspension surgery in conjunction with plastic surgery for the congenital left palpebral ptosis. Research by Rodden et al. [13] reported a nine-year-old male patient with DubS who developed nuclear cataracts at the age of 12, emphasizing the importance of addressing strabismus and cataracts in this syndrome and proposing the use of aphakic extended-wear contact lenses to treat ocular anomalies.

Dental care is another crucial aspect of treatment, as exemplified by our patient's management of dental hypoplasia and caries through pulpotomy, fluoride treatments, restorations, and the extraction of multiple teeth. Garrocho-Rangel et al. [23] describe the oral, craniofacial, and systemic features of a seven-year-old and 11-month-old Mexican boy with DubS in the mixed dentition stage. This patient presented with the absence of multiple teeth, caries, skeletal Class II malocclusion, maxillary space deficiency, and mandibular anterior crowding. He was treated with pit-and-fissure sealants, resin restorations, and the placement of functional orthopedic appliances. The authors emphasize the importance of both preventive and restorative measures to maintain dental health in these patients and note that dental aesthetic improvements can significantly enhance the patient's self-esteem.

A neurobehavioral assessment is fundamental in patients with DubS [11]. Mild to moderate intellectual disability is common [8], but severe intellectual disability has been observed in up to 7% of patients with DubS [11]. Parrish and Wilroy, Jr. [16] evaluated the psychological status of 10 individuals with DubS, where half of the children had low-average or average intelligence, while the other half had a range from mild to severe intellectual disability. The most affected cognitive functions were receptive vocabulary and fine motor development, followed by expressive vocabulary, with memory and reasoning being less impacted. Despite having average intelligence, our patient struggled with speech and language due to delayed intervention for his cleft palate and associated dental issues. Consequently, he was referred to the phoniatric and language therapy clinic, which yielded positive outcomes. The treatment with the speech therapist was comprehensive, addressing both unresolved phonological issues and the cognitive aspects related to the language delay. Notably, Huber et al. [11] emphasize the need to assess cognitive functionality, as well as speech and language, in all patients with DubS, as the majority of patients experience language development and speech difficulties arising from cognitive deficits or challenges in social skills. Tsukahara and Opitz [8] reported behavioral issues in 52 out of 141 patients with DubS, including hyperactivity, aggressiveness, and shyness. They also observed unique behavioral tendencies, such as an affinity for vibrations and musical beats, similar to those exhibited by our patient. Our case bears similarities to that reported by Chan et al. [15], which described a Chinese patient with DubS who was talkative and friendly. It is important to note that, in patients without behavioral issues as described in the literature, other psychological concerns such as low self-esteem may arise. Our patient experienced bullying at school and was abandoned by his father due to his condition, leading to low self-esteem. Behavioral development needs to be guided by a psychological team from an early age, as in our patients who received recurrent sessions. This approach is crucial for fostering social integration and addressing self-esteem concerns [11].

All members of this multidisciplinary team are obligated to conduct regular follow-up appointments, discuss various alternative treatments over the years, and assess the patient continuously throughout his life, including evaluating risks for malignancies to which these patients may be more susceptible.

Future directions in research 

While DubS remains a rare condition, the field of research surrounding its diagnosis, treatment, and long-term management is steadily evolving. Given the complexity and rarity of DubS, future studies are crucial to deepen our understanding of its genetic, clinical, and therapeutic dimensions. Several key areas warrant further investigation to improve the quality of care and patient outcomes in this patient population.

## Conclusions

Dubowitz syndrome is a rare condition that must be identified and treated promptly. As pediatricians are often the first point of contact for newborns, it is crucial for them to recognize the various clinical patterns associated with this syndrome and to refer patients to the appropriate specialists to initiate treatment early. Early intervention can significantly enhance developmental outcomes. A multidisciplinary approach is essential to address both the physical and psychological aspects of the condition; effective communication among all departments is necessary to implement a cohesive treatment plan that improves the patient's quality of life. By presenting this case and its successful multidisciplinary management, we aim to provide valuable insights that may inform future guidelines for the treatment of Dubowitz syndrome.

## Appendices

Appendix A

**Table 4:** Postoperative satisfaction scale

Score	Description
1	Regrets surgery
2	Not satisfied, but does not regret surgery
3	Acceptable, but not the smile expected; needs improvements
4	Satisfied, but needs minor adjustments
5	Completely satisfied
